# Effects of a dietary modification intervention on menstrual pain and urinary BPA levels: a single group clinical trial

**DOI:** 10.1186/s12905-021-01199-3

**Published:** 2021-02-09

**Authors:** SoMi Park, ChaeWeon Chung

**Affiliations:** 1 Department of Nursing, Wonju College of Medicine, Yonsei University, 20 Ilsan-ro, Wonju, Gangwon-do Korea; 2 College of Nursing, Research Institute of Nursing Science, Seoul National University, 103 Daehak-ro, Jongno-gu, Seoul, Korea

**Keywords:** Menstrual pain, Endocrine disruptor, Bisphenol A

## Abstract

**Background:**

Exposure to endocrine-disrupting chemicals (EDCs) occurs mainly through dietary intake. Due to current lifestyle trends, young people tend to consume fast food, to use disposable products, and to utilize convenient household items, all of which are major sources of EDCs. This study aimed to investigate the effects of a dietary modification intervention on menstrual pain and urinary bisphenol A (BPA) levels throughout three menstrual cycles in female college students who experienced severe menstrual pain. We also analyzed participants’ adherence to the intervention and examined whether their level of adherence was associated with differences in the effects of the intervention.

**Methods:**

A single-group pretest and repeated posttest experimental design was employed. Thirty female college students with a score of 5 or higher on a menstrual pain scale were recruited through convenience sampling. During three menstrual cycles, menstrual pain was scored on a 10-point scale after each cycle, and urinary BPA levels were measured from the first morning urine collected after each cycle. The intervention involved three components: small-group education, follow-up monitoring, and peer support via social network communication. Statistical analyses were conducted using Friedman one-way repeated-measure analysis of variance by ranks, non-parametric two-way analysis of variance, and the Wilcoxon signed-rank test as a post-hoc test.

**Results:**

The dietary modification intervention had significant effects on menstrual pain at all three time points of menstrual cycles (χ^2^ = 119.64, *p* = 0.000) and on urinary BPA levels until the 2^nd^ menstrual cycle (χ^2^ = 205.42, *p* = 0.000). Slightly fewer than half (43.3%) of the participants were highly adherent. Menstrual pain differed according to adherence level (F = 4.67, *p* = 0.032) and decreased over time through the third cycle post-intervention (F = 18.30, *p* = 0.000). Urinary BPA levels also decreased significantly (F = 7.94, *p* = 0.000), but did not differ according to adherence level.

**Conclusions:**

The dietary modification intervention was effective and sustainable for reducing menstrual pain and urinary BPA levels. Detailed information about EDCs and dietary experiences seemed to encourage the young women to become more concerned about EDCs and to perform self-protective actions. Further experimental research is suggested to examine the relationships of EDCs with various health indicators in women.

*Trial registration*: KCT0005472 at 2020-9-24 retrospectively registered.

## Background

Menstrual pain is one of the most common symptoms in women aged 20 years or younger without any pathological lesions in the pelvis, in which case it is referred to as primary dysmenorrhea [1]. The prevalence of primary dysmenorrhea varies across different countries from 85 to 93% [2–4], and 40% to 58% of those women reported experiencing a moderate to severe degree of pain [3, 5]. For this reason, one-third to one-half of women with menstrua; pain were absent from school or work at least once per cycle [6]. In addition, menstrual pain is accompanied by mood changes, poor appetite, nausea, vomiting, and dizziness [7], poor sleep quality [8], as well as diminished self-identity [9, 10]. Overall, painful menstruation negatively affects women’s physical and psychosocial health, with eventual impacts on their quality of life.

By the way, recent evidences have indicated endocrine-disrupting chemicals (EDCs) as one of the main causes of reproductive health problems, particularly in women. By mimicking estrogen and binding to the estrogen receptor [11], EDCs can induce menstrual abnormalities such as pain or heavy bleeding [12, 13], early menarche [14], precocious puberty [15], and infertility [16]. Nevertheless, women mostly take painkillers for symptom relief due to painful menstruation [17]. EDCs are mostly man-made compounds that are found in various materials in our environment. Since the negative impact of EDCs has emerged as a public health issue, the Healthy People 2020 initiative has established the following major goals for public health: to be aware of toxic substances and hazardous waste, to investigate and respond to disease, and to educate the public [18]. However, EDCs are handled under regulations for chemical agents in Korea, and no consensus exists regarding how to control consumers’ risk of exposure [19]. Among the various EDCs, bisphenol A (BPA) comprised the greatest proportion of household dust in data from South Korea, the United States of America (USA), Japan, and China; furthermore, the total amount of BPA exposure was higher in Korea, at 18.6 ng kg^−1^ body-weight^−1^ day^−1^, than in Japan (4.61), the USA (12.6), and China (15.8) [20].

Although humans are unconsciously exposed to toxic chemicals through different routes, exposure to EDCs mainly occurs by dietary intake, which accounts for more than 90% of the total chemical exposure [21]. Above all, BPA is a highly prevalent EDC that is contained in polycarbonate, plastics, and epoxy resins manufactured for food and beverage packaging [22, 23], then dietary intake contributes to 72.5% of BPA exposure overall, in most people [24]. In this regard, young people in reproductively active ages are thought to be vulnerable to BPA exposure as they are accustomed to a high-convenience lifestyle, tend to consume more fast foods and processed foods, and frequently use disposable products such as plastics and cans. Despite the growing and evident health impacts of EDCs on women, few interventions have attempted to attenuate their influence on women’s health problems. Although one study [25] tried a dietary intervention with canned and fresh foods, it only analyzed exposure to BPA in 20 men and women based on a follow-up 6 h after the meal. Another pilot intervention [26] which provided college-aged women with BPA-free products to use for the study, also confined its analysis to the reduction of urinary BPA levels during a 3-week intervention. Therefore, it was necessary to design a study to reflect women’s real-world circumstances, not conditional on the experiment, and to examine longer-term effects, particularly regarding women’s health indicators. This study presents a clinical trial that was designed to modify dietary lifestyle, along with an examination of its effects on menstrual pain and urinary BPA levels as a biochemical indicator.

Aim.

The specific aims of this study were; first, to examine the effects of a dietary modification intervention on menstrual pain and urinary BPA levels throughout three menstrual cycles in female college students; second, to evaluate how well the participants adhered to the intervention; third, to investigate whether their level of adherence was associated with differences in the effects of the intervention; and finally, to assess changes in menstrual pain and urinary BPA levels throughout three menstrual cycles.

## Methods

### Design

**Fig. 1:**
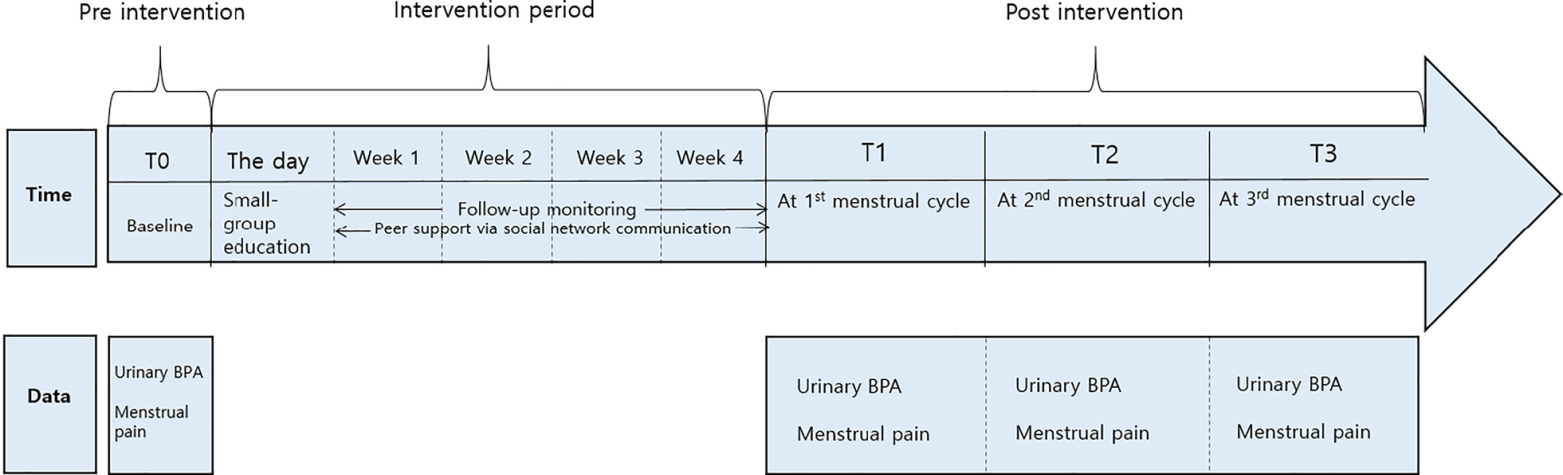
Research process and timeline of data collection

This study utilized a single-group pretest and repeated posttest experimental design. The dependent variables were measured three times at intervals of 4–6 weeks, depending upon each participant’s menstrual cycle (Fig. 1).

### Participants and sample size

Volunteers were recruited by a research assistant after flyers were posted throughout a college dormitory located in W city, Korea. Women were included if they (1) reported a score of 5 or higher on a menstrual pain scale of 10 points, (2) had not been diagnosed with any gynecologic diseases, (3) lived in a dormitory, and (4) voluntarily agreed to participate in the intervention involving collection of urine samples. Each woman with an intention to participate was informed by the research assistant to select one of three slots that would be within a week after their period, keeping in mind individual differences in the menstrual cycle. The participants were composed of nursing students in their sophomore and junior years, for whom participation in this trial was feasible and accessible, particularly for dropping off the fresh urine samples.

The study sample size was determined considering a one-tailed, Wilcoxon signed rank test, a type I error of 0.05, a statistical power of 0.80, and an effect size of 0.50. Using G*Power 3.1.9.2, this study required a total of 28 participants. Initially, 32 students were enrolled, anticipating a 10% attrition rate, and two students discontinued participation during the intervention period due to temporary withdrawal from school. Thus, there were 30 participants.

### The dietary modification intervention

The intervention involved dietary modifications targeting reduction of fast/processed food consumption. It was composed of three parts: (1) small-group education (2) follow-up monitoring, and (3) peer support via social network communication (Table 1).

**Table 1:** Structure of the dietary modification intervention for reduction of exposure to EDCs

Component	Contents		Methods
Small-group education (90 min)	Introduction (10 min.)	Welcome and information about the research process	Lecture with PPT
	Understanding of EDCs (30 min)	Definition of EDCs	Lecture with PPT
		Sources of EDCs in food, cooking, & containers	
		Mechanism of EDCs, women’s menstruation, & reproductive health	
	Self-appraisal (15 min)	Self-check of the degree of exposure to EDCs	Survey and comparison with peers
	Seeking solutions for minimizing exposure to EDCs (25 min.)	Identification of strategies to reduce BPA exposure in dietary habits	Lecture with PPT and discussion
	Self-contract (10 min.)	Making self-contract for the reduction of fast/processed food consumption	Writing a contract
Follow-up monitoring (4 weeks)	Monitoring by theresearch team	Confirmation of the checklist Feedback on practice Encouragement for empowering	Online messages from the research assistant in each of the three groups
Peer support via social network communication (4 weeks)	Communication with peers	Sharing experiences with peers	Social networking among the group members

EDCs, Endocrine-disrupting chemicals; PPT, PowerPoint

To ensure feasibility of the intervention, three groups were established and the participants selected one of the intervention slots depending upon their menstrual cycle; thus, each group was composed of 8, 9, and 13 participants, respectively. All participants received all three parts of the intervention.

The small-group education consisted of a 90-min session, which included information about EDCs and their impacts on women’s reproductive health problems, self-appraisal of exposure to EDCs, recommendations for restricting EDC intake, and a self-contract. The education was provided to each group in a seminar room available for audiovisual media services by a nurse interventionist who had a master’s degree and was blind to the study purpose. She was trained by the research team to deliver the educational content to each of the small groups. As human behavior is habitual and guided by automatic cognitive processes, knowledge and information were assumed to lead the women to act to protect themselves from exposure to EDCs.

Follow-up monitoring was conducted by a research assistant during the next 4 weeks covering the average interval of a menstrual cycle. Four-week follow-ups were assumed to be necessary to change the individual’s longstanding dietary habits [27, 28]. The participants in each group were monitored how well they adhered to the dietary modification intervention. Participants sent a weekly checklist to the assigned group assistant every Sunday. Then the research assistant replied with encouragement and praise after confirmation that participants had filled out the checklist. The checklist developed for this study was provided as Additional file 1.

Peer support via social network communication was composed of sharing their experiences and providing each other with emotional support within each small group. Peer support was planned based on a previous finding that peer recommendations positively influenced pro-environmental health behaviors in young women [28].

### Outcomes

The outcomes measured included menstrual pain and urinary BPA levels.

Menstrual pain was measured by a 10-point pain scale to indicate the maximum pain level experienced during their menstrual cycles. At baseline and after three consecutive menstrual cycles, women marked their perceived degree of pain on a visual analogue scale (1 = no pain to 10 = the worst possible pain) and returned it to the research team with the urine specimens. The menstrual pain recording sheet developed for this study was provided as Additional file 2.

Urinary BPA levels were measured because it has been used as a reliable method to monitor human intake of BPA [25, 29, 30]. The measurements were made four times, at baseline before the intervention and for three menstrual cycles after the intervention. To collect the urine samples, BPA-free specimen tubes labeled with the participant’s identification number and the order of the cycle were distributed to the participants at baseline data collection. Women were advised to obtain at least 2 mL of the first morning midstream urine, collected at their earliest convenience within one week after their period ended, and then to submit it to the research assistant that morning. Since all the participants resided in a campus dormitory, they dropped the samples at the office, which was about a 5-min walk from their dormitory, and it was then immediately frozen. As urinary BPA levels were not expected to show any significant difference according to the time lapsed from collection to analysis [31], the urine specimens were frozen until 30 samples were collected, after which the samples were sent to the Institute for Life & Environmental Technology for analysis. Urinary BPA levels were analyzed using high-performance liquid chromatography-mass spectrometry (a 6410b/Agilent; Agilent, Santa Clara, CA, USA) with corrected creatinine (Cr) values. The research team was blinded to the results until the specimens from the third cycle were analyzed to avoid possible interference with the participants.

### Data collection procedure

When the participants were invited to the educational component of the intervention, they were asked to provide a urine sample and to assess their menstrual pain as baseline data before starting the education session. After the session ended, they were provided three urine collection tubes and a sheet for recording menstrual pain for three consecutive menstrual cycles to take home. The sheet for recording menstrual pain and the urine collection tubes were delivered to the research office within a week after each of the three menstrual cycles during the study. Participants were also asked to fill out a checklist assessing their adherence to the dietary modification intervention every Sunday during the 4 weeks after the intervention. The participants e-mailed the checklist or sent it through a social networking service to the research assistant for each of the three groups. Study data were collected between December 2017 and May 2018.

### Statistical analysis

SAS version 9.4 and R version 3.6.3 were utilized with a significance level of less than 0.05. As descriptive statistics, median (minimum to maximum score) values were calculated. First, to examine the effects of the intervention on menstrual pain and urinary BPA levels at the first, second, and third menstrual cycles, the Friedman test was conducted. Second, to identify whether there were any significant differences in menstrual pain and urinary BPA levels from pre-intervention to the first, second, and third menstrual cycles according to adherence levels, the Wilcoxon signed-rank test was conducted. The Bonferroni correction was also applied to control for the increased risk of type I error due to multiple comparisons.

The degree of adherence was measured by the dietary modification checklist. It contained five items (cup noodles, instant food, delivery food with a disposable container, microwaving food in a plastic container, and using a paper cup for hot beverages and tea). Responses for each item were coded as 1 if a participant indicated that she had not engaged in the corresponding behavior at all, 2 for a frequency of 1–2 times/week, 3 for a frequency of 3–4 times/week, 4 for a frequency of 5–6 times/week, and 5 for a frequency of every day. When comparing scores between the baseline and the fourth week after the intervention, if intake or usage was reduced, it was coded as 1, whereas a score of 0 was assigned for items with the same or higher scores. The scores for changes in the five items were then summed to yield a score ranging from 0 to 5 points. Based on the median score, participants with a score of 3, 4, and 5 were classified as the high-adherence group and those with a score of 0, 1, and 2 were classified as the low-adherence group.

## Results

### Characteristics of the participants

The women were 22.1 ± 1.50 years old on average (ranged 21–27 years). Their mean age at menarche was 13.0 ± 1.56 years (ranged 10–17 years) and the mean interval of the cycles was 30.9 ± 4.20 days, ranging from 25 to 43 days.

### Effects of the dietary modification intervention at each of three time points from the baseline

The effects of the dietary modification intervention were significant on menstrual pain at all the three time points after intervention (χ^2^ = 119.64, *p* = 0.000). From the baseline, menstrual pain level was decreased significantly to the 1st menstrual cycle (Z = − 4.65, *p* = 0.000), the 2nd menstrual cycles (Z = − 4.66, *p* = 0.000), and the 3rd menstrual cycle (Z = − 4.56, *p* = 0.000). Meanwhile, urinary BPA level was significantly changed from the baseline (χ^2^ = 205.42, *p* = 0.000), though the effect of the intervention was found to be significant to the 1^st^ (Z = − 3.39, *p* = 0.001) and the 2nd menstrual cycles (Z = − 2.78, *p* = 0.005), but not to the 3^rd^ menstrual cycle (Z = − 1.08, *p* = 0.280) (Table 2).

**Table 2:** Effects of the dietary modification intervention at three time points from the baseline (N = 30)

Variables	T0	T1	T2	T3	χ^2^ (*p*)	T1-T0	T2-T0	T3-T0
	Median (min–max)					Z (*p*)		
Menstrual pain (1–10)	8.00 (6.00–10.00)	6.00 (1.00–8.00)	5.00 (3.00–8.00)	5.00 (2.00–10.00)	119.64 (0.000)	− 4.65 (0.000)	− 4.66 (0.000)	− 4.56 (0.000)
BPA (µg/g Cr.)	0.99 (0.22–3.99)	0.41 (0.06–1.42)	0.45 (0.07–1.42)	0.72 (0.08–2.45)	205.42 (0.000)	− 3.39 (0.001)	− 2.78 (0.005)	− 1.08 (0.280)

T0: baseline, before the intervention

T1, T2, and T3: at 1st, 2nd, and 3rd menstrual cycle after the intervention, respectively

### Level of adherence to the dietary modification intervention

When participants were analyzed by adherence level, the high-adherence group comprised 13 women (43.3%), while the other 17 women (56.7%) belonged to the low-adherence group.

### Effects the dietary modification intervention on menstrual pain and urinary BPA levels according to the level of adherence

Menstrual pain significantly differed between the high-adherence and the low-adherence groups (F = 4.67, *p* = 0.032) and the effects remained consistent over time (F = 18.30, *p* = 0.000). There was no interaction effect of group and time. Urinary BPA levels significantly changed over time (F = 7.94, *p* = 0.000), but did not differ by group, and the interaction effect of group and time was also found to be insignificant (F = 2.50, *p* = 0.063) (Table 3).

**Table 3:** Effects of the dietary modification intervention according to the level of adherence (N = 30)

Variable	Group	T0	T1	T2	T3	Source	F	*p*
		Median (min–max)						
Menstrual pain (1 ~ 10)	High-adherence(n = 13)	8.0 (6.0–10.0)	6.0 (3.0–8.0)	4.0 (3.0–7.0)	4.0 (3.0–7.0)	Group	4.67	0.032
	Low-adherence (n = 17)	8.0 (6.0–10.0)	6.0 (1.0–8.0)	6.0 (3.0–8.0)	6.0 (2.0–10.0)	Time Group*Time	18.30 1.08	0.000 0.359
BPA (µg/g Cr.)	High-adherence (n = 13)	1.4 (0.2–2.7)	0.4 (0.1–1.1)	0.5 (0.1–1.1)	0.6 (0.3–2.3)	Group	0.40	0.525
	Low-adherence (n = 17)	0.7 (0.3–4.0)	0.5 (0.1–1.4)	0.5 (0.1–1.4)	1.3 (0.1–2.9)	Time Group*Time	7.94 2.50	0.000 0.063

T0: baseline, before the intervention

T1, T2, and T3: at 1st, 2nd, and 3rd menstrual cycle after the intervention, respectively

### Changes in menstrual pain and urinary BPA levels in high-adherence and low-adherence groups

**Fig. 2:**
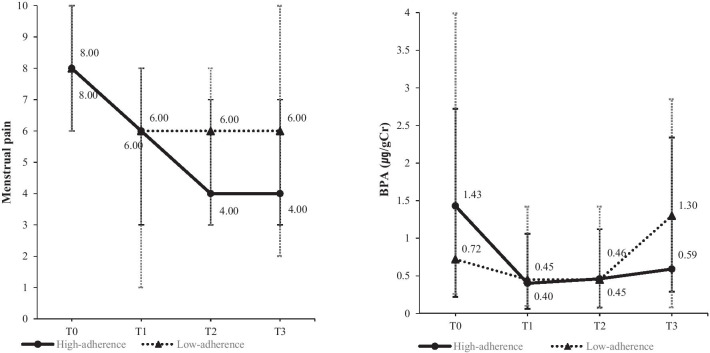
Changes in menstrual pain and urinary BPA levels in high-adherence and low-adherence group

Post-hoc testing showed that menstrual pain was alleviated significantly from baseline to all three menstrual cycles. These changes were significant from the baseline to the first cycle after intervention (W = − 217.50, *p* = 0.000), the second cycle (W = − 189.00, *p* = 0.000), and the third cycle (W = − 189.00, *p* = 0.000) (Fig. 2). Also, the urinary BPA levels changed from the baseline to the first menstrual cycle after intervention (W = − 165.00, *p* = 0.001) and to the second cycle (W = − 135.50, *p* = 0.020) (Fig. 2).

## Discussion

This study aimed to investigate the effects of a dietary modification intervention on menstrual pain and urinary BPA levels in female college students who experienced severe menstrual pain. We also assessed participants’ adherence to the intervention and examined whether their level of adherence was associated with differences in the effects of the intervention. The major finding was that the dietary modification intervention had significant effects on menstrual pain at all three time points of menstrual cycles and on urinary BPA levels until the second menstrual cycle. Menstrual pain differed according to adherence to lifestyle modifications and decreased over time through the third cycle post-intervention. Urinary BPA levels also decreased significantly, but did not differ according to adherence level. The interaction effects of group and time were insignificant for both menstrual pain and urinary BPA levels.

Despite the prevalence and the severity of menstrual pain, it has been treated as just a common and minor health condition that a person should simply endure instead of seeking medical advice. While various causes of menstrual pain are thought to exist, current evidence points to the physiological impacts of EDCs as a result of modern lifestyle changes [25, 32]. However, little evidence directly supports the relationship between environmental hormones and menstrual pain. A previous pilot study showed that a 3-week trial in which participants used BPA-free cosmetics and consumed food and water from glass containers led to a decrease in BPA levels [26], although it was suggested that further intervention studies should be conducted to confirm the results. Consistent with that finding, this study presents meaningful evidence that a dietary modification intervention was effective for decreasing urinary BPA levels throughout the second menstrual cycle (around 8 weeks) post-intervention and for alleviating menstrual pain up to the third menstrual cycle (around 12 weeks). The baseline BPA levels ranged from 0.22 to 3.99 μg/g Cr, and decreased to 0.06–1.42 μg/g Cr after the second cycle. In some sense, this change in BPA levels could be a useful cue to draw attention and to facilitate action in the young population instead of disseminating general guidelines. Therefore, this evidence regarding the relationship between exposure to EDCs and health problems could be utilized in the environmental movement to promote the consumption of healthy substances in people’s daily lives.

However, a pattern for BPA levels to increase after the second menstrual period was observed in the low-adherence group, and this finding is suggestive of the timing needed for reinforcement to sustain behavioral changes. Individual perceptions of vulnerability to a health risk and the seriousness of the health risk, as well as one’s belief in treatment effects, have an influence on whether behavioral changes are sustained [33]. Furthermore, as Lamptey et al. [34] found major misconceptions about intrauterine devices even among women users, individuals’ knowledge and information about EDCs and health risks could have influenced their level of behavioral maintanance. Therefore, visible evidence would also be expected to strengthen beliefs in self-driven behavioral and lifestyle modifications. It is presumed that the interaction effects were not significant because both groups of women were exposed to the same intervention with a small sample size.

Meanwhile, researchers and public health providers are frequently concerned with how well people adhere to the intervention or education given. In this study, the level of adherence was classified by using a dietary modification score between the baseline and the fourth week after the intervention, and 43.3% of the participants seemed to be relatively more motivated for change, whereas the others might not have felt that the modification was beneficial or faced barriers to continue the change. Considering a report that up to 40% of patients did not adhere adequately to physicians’ instructions [35], the adherence level of the non-patient participants in this study seemed to align with what could reasonably be expected; nonetheless, it is necessary to identify the factors influencing these young women’s adherence.

Based on the study outcomes, future interventions would consider targeting elementary-school girls through the school health system. Developing interventions that target elementary-school girls is logical because menstrual pain tends to occur after menarche [6], the age at menarche is becoming lower [36, 37], eating habits are becoming more convenience-oriented, and elementary-school girls are at a stage where they form habits in their daily life [38]. Therefore, it is critical for girls to form healthy dietary habits to protect themselves from exposure to EDCs starting at an early age. Parents and school health professionals likely provide the first line of guidance, and they need to be prepared for that responsibility. Policies and regulations to protect adolescents and young people from sources of EDCs exposure, such as cans and plastic bottles from vending machines available on campus, should be implemented in addition to social awareness and environmental policies of the government. Particularly, considering the finding that men and women were influenced by different negative and positive factors in even cigarette smoking participation and intensity [39], a gender-sensitive approach is thought to be more effective for providing education on EDCs and health in the future to both young men and women. In addition, the results of this study could be utilized in healthcare programs and education targeting risk factors such as obesity [40] and precocious puberty [15] which are thought to be associated with environmental hormones. Healthcare providers at schools, in communities, and at public health institutes should be prepared to play a key role in identifying hazardous chemicals, exposure routes, and illness sequelae, with the ultimate goal of protecting public health.

## Strengths and limitations

As it is impossible to control the dietary lifestyle of the participants in real life [41], the participants were classified into two groups according to their level of adherence; the low-adherence group served as a control, and the high-adherence group served as an experimental group, thereby minimizing this weakness of the study design. Additionally, repeated measures of the variables allowed us to observe the sustained effect of the intervention and to draw inferences regarding the timing for introducing a booster. Strengths of this study include the longer-term follow-up of outcomes, focusing on current women’s health symptoms and changes therein to assess the intervention effects, as well as the application of a behavioral intervention to real-world daily life.

However, the lack of randomization and the small sample size could still be limitations regarding the ability to infer causal relationships and to generalize the study outcomes. Moreover, measuring menstrual pain by self-reporting cannot exclude bias from an individual’s subjective judgment. For this single-group clinical trial, the participants were those who were interested in this study and who could drop off urine samples regularly within the boundaries of campus. Although it was not assumed that they had learned about EDCs and reproductive health in the nursing curriculum, this possibility could be a limitation to the generalizability of the study results to young women in the overall population.

## Conclusions

The dietary modification intervention was effective for reducing menstrual pain and urinary BPA levels. This empirical finding was meaningful as it was drawn from women’s own symptom experiences and evaluations, as well as urinary BPA levels as an objective measure. Despite the known health risks of EDCs, few actual guidelines for protective behaviors have been provided, whereas this study showed that detailed information about EDCs and dietary experiences seemed to encourage the young women to become more concerned about EDCs and to perform self-protective actions. Above all, the intervention was sustainable for the young women in terms of changing their usual lifestyle, including food consumption, containers, and usage of EDC sources in their daily lives. As this trial examined the validity and feasibility of a dietary modification intervention, it is expected to be utilized as the basis for a future randomized controlled trial with a larger sample size on the impact of interventions targeting EDCs on women’s health problems.

## Supplementary Information

**Additional file 1.** The checklist developed for this study.

**Additional file 2.** The menstrual pain recording sheet developed for this study.

## Data Availability

The datasets used and analysed during the current study are available from the corresponding aurhor on reasonable request.

## Abbreviations

EDCs: Endocrine-disrupting chemicals
BPA: Bisphenol A
SAS: Statistical analysis system
Cr: Creatinine
Min: Minimum
Max: Maximum
