# Supplementary material for: Effects of a dietary modification intervention on menstrual pain and urinary BPA levels: a single group clinical trial
Source: BMC Womens Health. 2021 Feb 9;21:58. doi: 10.1186/s12905-021-01199-3 (PMC7871389; doi:10.1186/s12905-021-01199-3)
Supplement: Supplementary file 1 — Additional file 1. The checklist developed for this study. [file 12905_2021_1199_MOESM1_ESM.pdf]

Menstrual Pain Recording Sheet

Please circle the number indicating the degree of pain that you experience during the period.

1	2	3	4	5	6	7	8	9	10
No pain									The worst possible pain
