# Supplementary material for: Effects of a dietary modification intervention on menstrual pain and urinary BPA levels: a single group clinical trial
Source: BMC Womens Health. 2021 Feb 9;21:58. doi: 10.1186/s12905-021-01199-3 (PMC7871389; doi:10.1186/s12905-021-01199-3)
Supplement: Supplementary file 2 — Additional file 2. The menstrual pain recording sheet developed for this study. [file 12905_2021_1199_MOESM2_ESM.pdf]

Diet Checklist

Please write the number of times you had each of the five items. This weekly form will be provided for 4 weeks.

	**Mon**	**Tue**	**Wed**	**Thu**	**Fri**	**Sat**	**Sun**	**Total**
1. Cup noodles								
2. Instant food								
3. Delivery food with a disposable container								
4. Microwaving food in a plastic container								
5. A paper cup for hot beverages and tea								
